# Diabetic Peripheral Neuropathy in Ethiopia: A Systematic Review and Meta-Analysis

**DOI:** 10.1155/2021/5304124

**Published:** 2021-02-04

**Authors:** Degena Bahrey Tadesse, Gebremeskel Tukue Gebrewahd, Abrha Hailay, Woldu Aberhe, Guesh Mebrahtom, Kidane Zereabruk, Guesh Gebreayezgi, Teklewoini Mariye, Teklehaimanot Gereziher Haile, Gebreamlak Gebremedhn Gebremeskel, Gebre Teklemariam Demoz

**Affiliations:** ^1^Department of Adult Health Nursing, School of Nursing, Aksum University, Aksum, Ethiopia; ^2^Department of Emergency Nursing, School of Nursing, Aksum University, Aksum, Ethiopia; ^3^Department of Epidemiology, School of Public Health, Aksum University, Aksum, Ethiopia; ^4^Department of Maternal and Neonatal Nursing, School of Public Health, Aksum University, Aksum, Ethiopia; ^5^Department of Clinical Pharmacy, School of Pharmacy, Aksum University, Aksum, Ethiopia

## Abstract

**Background:**

Currently, diabetic peripheral neuropathy (DPN) is one of the most severe complications of diabetes mellitus (DM). Despite the seriousness of this problem, limited evidence is available on the prevalence of diabetic peripheral neuropathy among patients with diabetes mellitus in Ethiopia. In Ethiopia, there were no updated studies that estimate the national prevalence of DPN. Hence, this systematic review and meta-analysis provided a national prevalence of diabetic peripheral neuropathy among patients with diabetes mellitus in Ethiopia.

**Methods:**

This study was submitted for registration with the International Prospective Register of Systematic Reviews (PROSPERO) in March 2020 and accepted with the registration number CRD42020173831. Different database searching engines were searched online to retrieve related articles, including PubMed, Scopus, Google Scholar, African Journals Online, World Health Organization (WHO) Afro Library, and Cochrane Review. The reviewers used the Preferred Reporting Items for Systematic Review and Meta-Analysis (PRISMA) guideline in the reviewing process. In this systematic review and meta-analysis, all published and unpublished articles were analyzed. The reviewers used the random effects model to estimate the pooled prevalence of diabetic peripheral neuropathy among diabetes mellitus patients. The reviewers conducted the statistical analysis using the R version 3.5.3 and RStudio version 1.2.5033 software for Windows. The reviewers evaluated the heterogeneity across the included studies by the inconsistency index (*I*^2^). The reviewers examined the publication bias by the funnel plot.

**Results:**

The search of the databases produced 245 papers. After checking the inclusion and exclusion criteria, 38 articles with 14029 total patients with diabetes mellitus were found suitable for the review. Except for three (retrospective cohort study), all studies were cross-sectional. The overall pooled prevalence of diabetic peripheral neuropathy was 22% (95% CI 18% to 26%). The subgroup analysis of diabetic peripheral neuropathy among patients with diabetes in the different regions was 23% (95% CI 17% to 29%) in Addis Ababa, 27% (95% CI 16% to 38%) in Oromia, 16% (95% CI 14% to 18%) in South nation and nationalities, and 15% (95% CI 6% to 24%) in Amhara.

**Conclusions:**

More than one-fifth of patients with diabetes have diabetic peripheral neuropathy. According to this study, the prevalence of diabetic peripheral neuropathy in Ethiopia is considerably high. This evidence suggests that attention should be given to patients with diabetes in monitoring patients' blood glucose.

## 1. Introduction

Currently, half a billion people are living with diabetes worldwide and will increase by 578 million (25%) in 2030 and 700 million (51%) in 2045 [1].

Diabetes mellitus (DM) is a metabolic disorder that can predispose to diseases of the heart, blood vessels, lungs, kidneys, and nerves. Such damage may result in a decreased blood flow to the feet, which is coupled with neuropathy to the nerves—raising the risk of foot ulcers, inflammation, and subsequent limb amputation [2].

One of the most severe complications of DM is diabetic polyneuropathy (DPN). The lifetime prevalence of DPN was 50% [3–5]. DPN is associated with a significant reduction in quality of life and poses treatment challenges to the practicing physician [4]. It is also the cause of impairment due to foot ulceration and amputation, disruption of the gait, and damage involved with dropping. About 20 to 30 percent of DPN patients suffer from neuropathic pain [4–6].

The average estimated expense for diabetes medication, health insurance of DPN, and debilitating DPN is $6632, $12492, and $30755, respectively [7].

In Ethiopia, the prevalence of DPN is estimated to be in the range of 1.9–53.6% that shows a difference across different geographical settings and time [8, 9]. Many factors contribute to this huge difference, including study quality, study design, ethnic differences, and different diagnostic methods. Reliable estimates of DPN prevalence are required to enhance awareness of the prevention and management of DPN in patients with diabetes. Therefore, the primary purpose of this systematic review and meta-analysis is to determine the prevalence of DPN among patients with diabetes mellitus in Ethiopia.

## 2. Materials and Methods

### 2.1. Setting

Ethiopia is an East African country containing ten regions, namely, Tigray; Afar; Amhara; Oromia; Somali; Benishangul-Gumuz; Southern Nations, Nationalities, and People's Region (SNNPR), Gambella; Sidama Harari; and two urban administrative states (Addis Ababa City administration and Dire Dawa City administration).

### 2.2. Search Strategy and Information Sources

A search strategy was implemented using electronic databases (PubMed/MEDLINE, Embase, Google Scholar, Web of Science, Cochran Library, Africa-Wide Information, World Health Organization (WHO) Afro Library, and African Index Medicus) from inception to October 2020.

The reviewers have checked the presence of a precursor systematic review and protocol on the topic of interest via searching for different databases. The included databases were the Cochrane Database of Systematic Reviews, Joanna Briggs Institute Database of Systematic Review and Implementation Reports (JBI-DSRIR), the national health center review and dissemination database, health technology assessment (HTA), the Campbell Collaboration Library, and Evidence for Policy and Practice Information (EPPI-Centre).

The reviewers have developed a literature search technique using the headings of the medical subject headings (Met) and Boolean (AND/OR) operator. The reviewers have used the combination of key terms “DPN”, “Diabetic peripheral neuropathy”, “Diabetes mellitus”, “Diabetic complication”, “Macro and micro-vascular diabetic complication”, “diabetic polyneuropathy”, “Ethiopia”, “systematic review”, and protocols. The search from the above databases confirmed that there was no systematic review and/or protocol on the topic of interest.

### 2.3. Data Extraction, Selection, and Process

Based on the inclusion and exclusion criteria, the reviewers have developed a tool to guide the screening and selection process. This tool was piloted and revised before data extraction begins. The search results were first uploaded to the EndNote software to remove duplicates.

Two blind and independent reviewers extracted the data using a preconceived and standardized data collection format. The two reviewers (DBT and GGG) screened the titles, abstracts, and full-text search results to identify potentially eligible studies. Where necessary, the authors were contacted for additional information to confirm the eligibility of the studies. Disagreements between the reviewers were resolved by discussion with the help of a third independent reviewer. Where there is missing information, the reviewers contacted the corresponding author of the study to request the missing data. A maximum of three emails was sent to the corresponding author of the retrieved studies to request additional information before excluding the article. For studies appearing in more than one publication, we considered the most recent and comprehensive studies and those with the largest sample size. For surveys appearing in one article with multiple surveys conducted at different time points, we treated each as a separate study.

The components of the data extraction format included the information on the year of publication, region, authors, and country; setting, objective, and study design; diagnostic criteria of DPN; prevalence; or incidence of DPN.

### 2.4. Criteria for Considering Studies for the Review

#### 2.4.1. Inclusion Criteria

The inclusion criteria are as follows:

*Design*: all observational studies.

*Population*: study participants of at least 18 years of age with type 1 or 2 DM.

*Publication status*: published and unpublished studies.

*Settings*: all health institution-based studies.

*Language*: articles in the English language.

*Publication or report year*: studies conducted from 1976 to 2020.

*Method of diagnosis*: all studies, regardless of the method used for diagnosing DPN.

*Intervention(s)/exposure(s)*: patients taking any form of antidiabetic medication.

*Outcome*: the primary outcome is the prevalence of DPN among diabetes mellitus patients in Ethiopia. It is defined by international consensus guidelines as “the presence of symptoms and/or signs of peripheral nerve dysfunction in people with diabetes after exclusion of other causes” [10, 11].

#### 2.4.2. Exclusion Criteria

The exclusion criteria are as follows: case report and case series studies, studies that lack relevant data needed to compute the prevalence of DPN, studies on pregnancy-related diabetes mellitus, and studies on children and adolescents < 18 years.

### 2.5. Risk of Bias and Quality Assessment of the Included Studies

Methodological quality and risk of bias assessments were performed by two reviewers (DBT and GGG), blindly and independently. The reviewers maintained the blinding reviewing method using the Covidence software that allows/obligates each reviewer to work without knowing the other reviewer's choice. This helps to diminish errors and the risk of bias in the selection of the studies. Disagreements between the reviewers were resolved by discussion and where necessary involving a third author.

For each included study, we estimated the precision (*C*) or margin of error, considering the sample size (SS) and the observed prevalence (*p*) of DPN using the formula:

(1) $$\begin{array}{c} \text{SS}=\frac{z^{2}*p*\left(1-p\right)}{d^{2}}, \end{array}$$

where *z* was the *z* value fixed at 1.96 across studies (corresponding to 95% confidence interval). The desirable margin of error is 5% (0.05) or lower.

The methodological quality was evaluated using the Newcastle-Ottawa Scale. The scale is primarily formulated by a star allocation system, assigning a maximum of 10 stars for the risk of bias in three areas: a selection of study groups (4 or 5 stars), comparability of groups (2 stars), and ascertainment of the outcome of interest or the exposure (3 stars). No validation study provides a cutoff score for rating low-quality studies. We arbitrarily established 0–3, 4–6, and 7–10 stars considered at high, moderate, and low risk of bias, respectively [12].

### 2.6. Data Analysis and Presentation of Results

The reviewers have registered this review in PROSPERO with the registration number (CRD42020173831), and the Preferred Reporting Items for Systematic Reviews and Meta-Analyses (PRISMA) guidelines have been used to report the results [13]. The data were analyzed using the R version 3.5.3 and RStudio version 1.2.5003 software. The pooled prevalence of diabetic peripheral neuropathy was estimated using forest plots and the extent of statistical heterogeneity between studies. Statistical heterogeneity was assessed using the standard chi-squared test (Cochran *Q* test) and quantified by calculating the *I*^2^ statistics (with values of 25%, 50%, and 75% assumed to be representative of low, medium, and high heterogeneity, respectively) [14]. There was clinical heterogeneity between the included studies. Consequently, we used a random effects meta-analysis to estimate the overall pooled prevalence of DPN in Ethiopia. The reviewers used a funnel plot to evaluate publication bias and the 95% confidence interval (CI) to describe the results.

## 3. Results

### 3.1. Screening Flow

**Figure 1:**
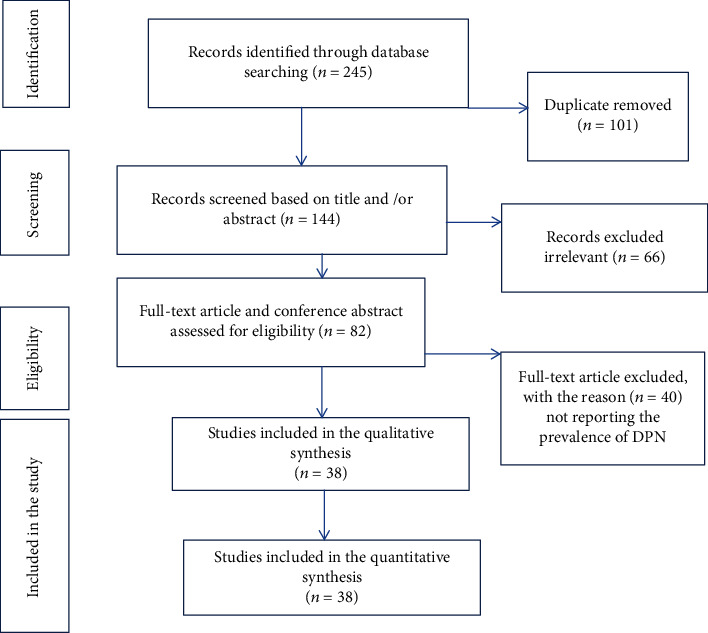
Flow chart diagram showing the selection of articles for systemic review and meta-analysis of DPN among patients with diabetes in Ethiopia, 2020.

Figure 1 is a flow diagram outlining the process of identification and selection of the included studies. The included databases and number of included studies thereof were PubMed (63), Scopus (46), Google Scholar (107), and the World Health Organization (WHO) Afro Library (29). Of these studies, 101 are duplicates and removed. Subsequently, we screened 144 titles and abstracts and excluded 66 irrelevant papers. Then, based on the predefined criteria and quality assessment, 38 full-text articles with 14029 total patients with diabetes were included in this systematic review and meta-analysis. The detailed steps of the screening process are shown in a PRISMA flow chart (Figure 1).

### 3.2. Study Characteristics

More than one-third (16, 42.1%) of the studies were from studies conducted in the capital city of Ethiopia (Addis Ababa), 9 (28.9%) in Oromia, 7 (18.2%) in Amhara, 2 (5.26%) in South Nation and Nationalities, and 1 (2.6%) in Tigray. Except for three studies (cohort), all the rest were cross-sectional. Of these studies, six studies were among patients with type 2 diabetes mellitus, and the rest were conducted on type 1 and type 2 diabetes mellitus. The quality of each primary study assessed using the Newcastle-Ottawa Scale shows no considerable risk. Therefore, all the included studies considered in this systematic review and meta-analysis were with a low risk of bias (Table 1).

### 3.3. The Pooled Prevalence of DPN

**Figure 2:**
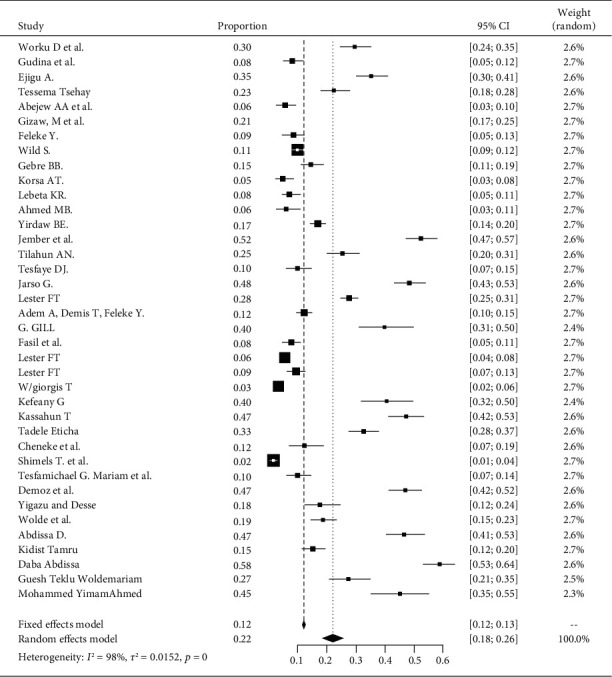
Forest plot for the pooled prevalence of DPN from 38 observational studies.

The pooled prevalence of DPN among patients with diabetes in Ethiopia is 22% (95% CI 18-26%). The heterogeneity test shown in *I*^2^ with 98% indicates high heterogeneity. The prevalence of diabetic peripheral neuropathy is varying widely in the studies. This heterogeneity might be due to differences in the diagnostic criteria employed and the different defining criteria of DPN used (Figure 2).

**Figure 3:**
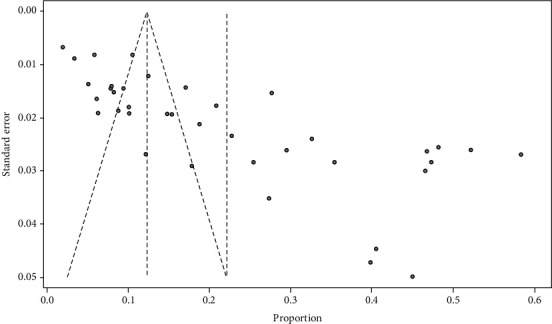
Funnel plot showing evidence of publication bias across studies.

A funnel test was used to test for publication bias. And it showed the presence of publication bias. Therefore, there are unpublished data that can modify the prevalence of DPN (Figure 3).

### 3.4. Subgroup Analysis of DPN by Region of the Country

**Figure 4:**
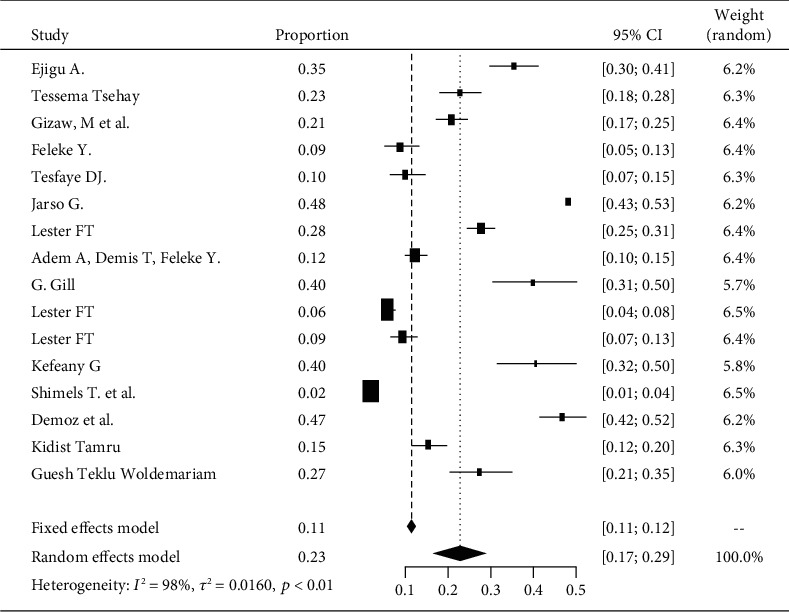
Forest plot of DPN in Addis Ababa.

Based on the subgroup analysis of DPN among patients with diabetes attending hospitals in Addis Ababa, the pooled point estimate was 23% (with 95% CI; 17 to 29) (Figure 4).

**Figure 5:**
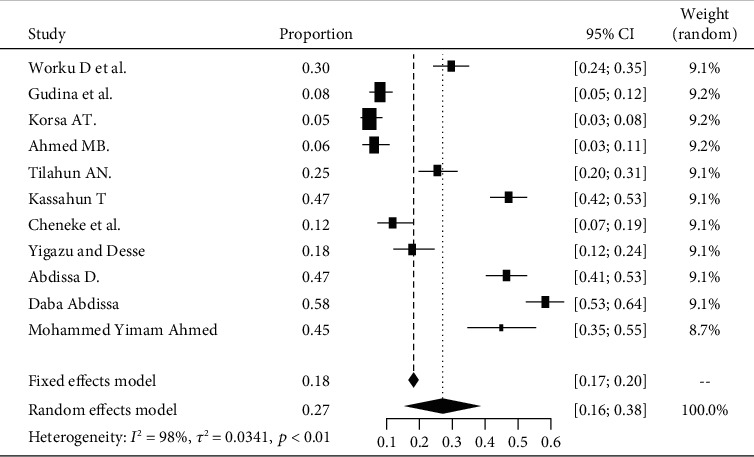
Forest plot of DPN in the Oromia region.

Based on a subgroup analysis of DPN among patients with diabetes attending Oromia's hospitals, the pooled point estimate was 27% (with 95% CI; 16 to 38) (Figure 5).

**Figure 6:**
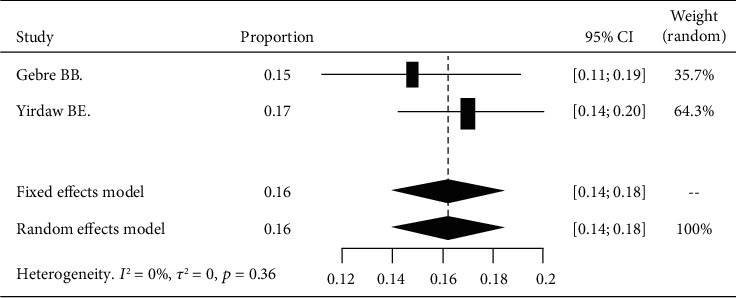
Forest plot of DPN in the south region.

Based on the subgroup analysis of DPN among patients with diabetes attending South Nation and Nationalities' hospitals, the pooled point estimate was 16% (with 95% CI; 14 to 18) (Figure 6).

**Figure 7:**
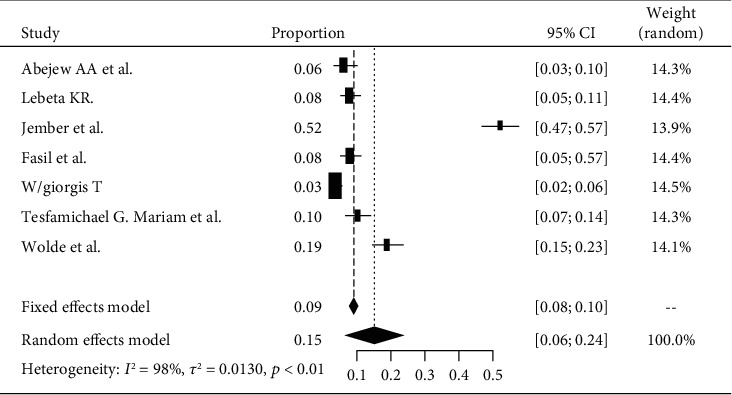
Forest plot of DPN in the Amhara region.

Based on the subgroup analysis of DPN among patients with diabetes attending Amhara's hospitals, the pooled point estimate was 15% (with 95% CI; 6 to 24) (Figure 7).

## 4. Discussion

The reviewers have performed the present systematic review and meta-analysis to produce pooled estimates of nationwide results of peripheral neuropathy in Ethiopia among patients with diabetes. This review emphasized the burden of peripheral neuropathy among patients with diabetes in Ethiopia for a better understanding of the medical condition that can help in mitigating the problem of peripheral neuropathy in the country.

This meta-analysis showed the pooled estimate of diabetic peripheral neuropathy in Ethiopia to be standing at 22% (with 95% CI: 18-26). Individually, there was a variety in the prevalence of peripheral neuropathy in the studies included in our work which ranged from 1.9% as reported by Jember et al. [9] to 53.6% by Abdissa [47]. The reason for these variations could be due to the types of diabetes and assessment methods. The estimates of DPN are more common in patients with type 2 diabetes compared to those with type 1 diabetes (53% in type 2 diabetes) in the meta-analysis reported from Iran [50] versus type 1 (28.2% in type 1) [51]. As we estimated both in one, this truth is supported by one systematic review, reported in its subgroup analysis; the higher prevalence of peripheral neuropathy was lesser in patients with type 2 diabetes (38.8%) than in patients with type 1 diabetes (17.5%) [52]. The overall pooled prevalence of peripheral neuropathy in this study (22%) was consistent with a study reported from Arab countries that had a pooled prevalence of diabetic peripheral neuropathy of 18% (with a 95% CI: 0.09-0.34) [52]. Similarly, our finding was also comparable with a systematic review and meta-analysis reported from Oceania (23.2%, 95% CI: 2.68-76) [53]. This similarity might be due to the type of diabetes in our work conducted on both type 1 and type 2 diabetes, and the meta-analysis reported from Iran [52] focused on studies which included only patients with type 1 diabetes.

On the other hand, the result found in this study is lower as compared to other previous systematic review and meta-analysis studies that estimated the prevalence of peripheral neuropathy in patients with diabetes: 46% from the African region [54], 53% from Iran [50], and 31.6%, 32.2%, and 48.1% from America, Asia, and Europe, respectively, with an overall prevalence of 35.7% [53]. Similarly, the result was smaller than the results found in other meta-analyses conducted among patients with type 1 DM which showed a prevalence of 28.2% in Iran [51] and 30% in studies conducted in multination [55]. Our findings were higher compared with a worldwide estimate of DPN prevalence among patients with diabetes (8.1%–12.2%) [56].

To look at the heterogeneity of the studies included in this review, we conducted subgroup analysis by regions, and we found that a study done in Addis Ababa had the highest pooled point estimate of peripheral neuropathy in patients with diabetes, accounting for 23% (95% CI: 16, 30), and the lowest prevalence was reported from the Amhara region (15%, 95% CI: 6, 24). This discrepancy could be due to the difference in health care settings such as tertiary and primary health care that may cause a difference in screening and assessment practice and quality of service for diabetes patients with peripheral neuropathy. Some health care facilities may be enriched with the specialty that works more on better assessment or good practice on prevention strategies or well found with diagnostic instruments and severity of diabetes. Moreover, it may also be because there are no uniform assessment criteria or diagnostic method in clinical practice in all health care settings or due to the use by different clinicians of their unique clinical expertise in examining and individualizing their patients.

Our findings from the meta-analysis have implications in clinical practice as it can contribute to giving attention to the prevention and care of patients with diabetes. This pooled point of estimates for peripheral neuropathy in patients with diabetes provides updated evidence to advance prevention strategy, serves as key indicators of patient safety, and reflects the quality of the health care service and appropriate treatment strategy for peripheral neuropathy in patients with diabetes. The finding of the prevalence of diabetic peripheral neuropathy improves with good glucose control; use of adherence interventions, such as patient education and counseling on how to self-monitor blood glucose; and lifestyle modification interventions, such as exercise, weight reduction, and healthy diet. Therefore, the glycemic control strategy should be kept in mind that in addition to prescribing appropriate antidiabetic medicines and diabetes care practice, they need to include resources that help patients overcome individual challenges to reduce the development of such chronic diabetic complications using self-care practice. The implication of this study, particularly the pronounced variation between studies (1.9% to 53.6%), reflects patients with diabetes which require developing standards for management and implementing endorsed guidelines for clinical practice.

## 5. Limitations of This Study

We conducted this review with rigor and standards of the art. So far, this is the first systematic review and meta-analysis that draws a clear picture of the pooled point of prevalence for peripheral neuropathy in patients with diabetes in Ethiopia. However, our work has certain limitations. Among these limitations are studies that did not describe the type of diabetes, and this could induce errors by finding untrue substantial associations when combining such studies affected by confounding. It was challenging to do a subgroup analysis for studies conducted among patients with type 1 and type 2 diabetes and those subjected to a high degree of heterogeneity among studies as we could not report by type of diabetes. But the random effects model was used to obtain the pooled results that can minimize such heterogeneity among studies. Additionally, the defining criteria for DPN differed from one to another study. This could also affect the results of the meta-analysis with far-reaching clinical heterogeneity across studies. The presence of publication bias was an additional limitation.

## 6. Conclusion

Our work systematically summarises the prevalence of peripheral neuropathy among patients with diabetes in Ethiopia that revealed that more than one-fifth of the patients with diabetes had developed peripheral neuropathy. Thus, our finding suggests that the prevalence of peripheral neuropathy is high at the national level but still lower than other previous studies reported from different countries. The Ministry of Health, health policymakers, clinicians, and other health care providers should strengthen the quality of health care service for patients with diabetes to reduce the development of peripheral neuropathy. The Ministry of Health should develop a context-based intervention and preventive strategies to reduce the burden of diabetes-related complications, particularly DPN.

## Figures and Tables

**Table 1:** Characteristics of studies included in the systematic review and meta-analysis of the prevalence of DPN among adult patients with diabetes in Ethiopia, 2020.

Author	Publication year	Data collection year	Study region	Types of hospital	Types of DM	SD	Sample size			Number of patients with DPN by type of DM			Prevalence (%)	Methods for detecting DPN	Quality assessment (based on NOS)
							Type I	Type II	Total	Type I	Type II	Total			
Worku et al. [15]	2010	October 2008	Oromia	Referral	Types I and II	CS	116	189	305	19	71	90	29.5	MNSI assessment	8
Gudina et al. [16]	2011	From August to November 2009	Oromia	Referral	Types I and II	CS	117	212	329	NM	NM	27	8.2	DNS instrument	7
Ejigu [17]	2000	September 1996 to July 1997	AA	Referral	Types I and II	RDS	112	171	283	25	75	100	35.3	DNE instrument	9
Tsehay et al. [18]	2014	From May to June 2014	AA	Referral	Type II	CS			321		73	73	22.7	DNE instrument	7
Abejew et al. [19]	2015	From April to May 2013	Amhara	Referral	Types I and II	CS	NM	NM	216	NM	NM	13	6	DNS instrument	6
Gizaw et al. [20]	2015	Between January 2010 and December 2013	AA	Referral	Types I and II	RDS	122	401	523	20	89	109	20.8	DNE instrument	8
Feleke and Enquselassie [21]	2005	From February 2002 to July 2003	AA	Mixed	Types I and II	CS	50	179	229	NM	NM	20	10	DNE instrument	7
Wild et al. [22]	2000	1993	Different regions	Mixed	Type II	CS	—	1386	1386	—	146	146	10.5	MNSI assessment	8
Gebre and Assefa [23]	2019	From April 1 to April 2019	SNNPR	General	Types I and II	CS	160	178	338	NM	NM	50	14.8	NSS, NDS	7
Korsa et al. [24]	2019	Between March and April 2016	Oromia	Mixed	Types I and II	CS	148	109	257	NM	NM	8	3.11	NSS, NDS	6
Lebeta et al. [25]	2017	From April to May 2015	Amara	Referral	Type II	CS	—	344	344	—	344	27	7.8	NSS, NDS	9
Ahmed et al. [26]	2018	From July to August 2015	Oromia	Referral	Types I and II	CS	70	90	160	NM	NM	10	6.3	DNS instrument	7
Fiseha and Belete[27]	2018	From January 2008 to August 2015	SNNPR	Referral	Types I and II	CO	187	495	682	NM	NM	116	17	DNS instrument	8
Jember et al. [9]	2017	From February 2016 to June 30 2016	Amara	Referral	Types I and II	CS	164	204	368	84	108	192	52.2	DNS instrument	8
Tilahun et al. [28]	2017	October to December of 2015	Oromia	Referral	Types I and II	CS	98	138	236	NM	NM	60	25.4	NSS, NDS	7
Tesfaye [29]	2014	From October to November 2013	AA	Referral	Types I and II	CS	56	191	247	NM	NM	25	10.1	Clinical examination	6
Jarso et al. [30]	2011	December 2009 to February 2010	AA	Referral	Types I and II	CS	104	280	384	86	99	185	48.2	DNS instrument	9
Lester [31]	1984	1982	AA	Referral	Types I and II	CS	NM	NM	849	NM	NM	235	27.8	DNS instrument	8
Fiseha and Belete [27]	2011	2010	AA	Referral	Types I and II	CS	NM	NM	724	NM	NM	90	12.4	Clinical examination	9
Gill et al. [32]	2008	2007	AA	Referral	Types I and II	CS	NM	NM	108	NM	NM	43	41	Clinical examination	7
Fasil et al. [33]	2019	February–March 2017	Amara	Referral	Types I and II	CS	NM	NM	367	NM	NM	29	7.9	DNS assessment	8
Lester [34]	1983	1983	AA	Referral	Types I and II	CS	NM	NM	809	NM	NM	47	5.8	DNE instrument	7
Lester et al. [35]	1976	1976	AA	Referral	Types I and II	CS	NM	NM	404	NM	NM	38	9.4	DNS assessment	6
Teshager [36]	2016	March–April 2016	Amhara	Referral	Types I and II	CS	178	238	416	NM	NM	14	3.4	DNS assessment	9
Kefeany [37]	2017	March–May 2017	AA	Referral	Type II	CS	—	121	121	—	49	49	40.5	NSS, NDS	8
Kassahun et al. [38]	2016	February–April 2014	Oromia	Referral	Type II	CS	—	309	309	—	146	146	47.2	DNE instrument	8
Eticha et al. [39]	2016	February 2015	Tigray	Referral	Type II	CS	—	384	384	—	125	125	32.6	NSS, NDS	7
Cheneke et al. [40]	2016	From May to July 2012	Oromia	Referral	Types I and II	CS	NM	NM	148	NM	NM	18	12.2	Clinical examination	6
Shiels et al. [8]	2018	From October 2016 to January 2017	AA	Referral (police)	Type II	CS	—	414	414	—	8	8	1.9	NSS, NDS	8
Mariam et al. [41]	2017	From March to April 2016	Amhara	Referral	Types I and II	CS	110	169	279	NM	NM	28	10	NSS, NDS	7
Demoz et al. [42]	2019	From August 2017 to July 2018	AA	Referral	Type II	CS	—	357	357	—	167	167	46.8	Clinical examination	7
Yigazu and Deese [43]	2017	From February to March 2016	Oromia	General	Type II	CS	—	174	174	—	31	31	17.8	Clinical examination	8
Woldu et al. [44]	2018	March 2017	Amhara	Referral	Type II	CS	—	341	341	—	64	64	18.8	DNE instrument	9
Abdissa et al. [45]	2019	June to August 2019	Oromia	Referral	Types I and II	CS	48	229	277	NM	NM	129	46.6	NSS, NDS	9
Tamura et al. [46]	2020	March 1 to April 28, 2019	AA	Referral	Type II	CS	—	346	346	—	53	53	15.3	MNSI assessment	7
Abdissa et al. [47]	2020	September to November 2019	Oromia	Referral	Type II	CS	—	336	336	—	196	196	53.6	DNS instrument	8
Woldemariam et al. [48]	2020	March to May 2019	AA	Referral	Types I and II	CS	NM	NM	161	NM	NM	44	27.3	DNE instrument	9
YimamAhmed et al. [49]	2020	February to March 2019	Oromia	Referral	Types I and II	CS	24	76	100	NM	NM	45	45	DNE instrument	9

AA: Addis Ababa; SNNPR: South Nation Nationalities and People Representative; NM: not mentioned; CS: cross-sectional; SD: study design; RDS: retrospective descriptive study; CO: cohort; NOS: Newcastle-Ottawa Scale; NSS: neuropathy symptom. The score: DNS: diabetic neuropathy symptom; DNE: diabetic neuropathy examination; MNSI: Michigan Neuropathy Screening Instrument.

## Abbreviations

DM: Diabetes mellitus
DPN: Diabetic peripheral neuropathy
PRISMA-P: Preferred Reporting Items for Systematic reviews and Meta-Analysis protocol.

## Supplementary Materials

Supplementary Materials The supplementary material includes the appendix about the search strategy and information sources.
