# Supplementary material for: Diabetic Peripheral Neuropathy in Ethiopia: A Systematic Review and Meta-Analysis
Source: J Diabetes Res. 2021 Feb 4;2021:5304124. doi: 10.1155/2021/5304124 (PMC7880716; doi:10.1155/2021/5304124)
Supplement: Supplementary Materials — The supplementary material includes the appendix about the search strategy and information sources. [file 5304124.f1.docx]

**Appendix**

## Search strategy and information sources

A search strategy was implemented using Electronic databases (Pubmed/MEDLINE, Embase, Google Scholar, Web of Science, Cochran library, Africa Wide Information, World Health Organization (WHO) afro library, and Africa Index Medicus) from inception to 19 January 2020.

The presence of precursor systematic review and/or protocol on the topic of interest was checked via searching different databases. The databases searched include the Cochrane database of a systematic review, Joanna Briggs Institute database of a systematic review and implementation reports (JBI-DSRIR), the national health center review and dissemination database, health technology assessment-HTA, the Campbell collaboration library and evidence for policy and practice information (EPPI-centre).

The literature search technique was developed using the headings of the medical subject headings (Met) and BOOLEAN (AND/OR) operator was used.  The combination of key terms including “DPN”, “Diabetic peripheral neuropathy”, “Diabetes mellitus”, "Diabetic

complication", "Macro and microvascular diabetic complication", “diabetic polyneuropathy” “Ethiopia, "systematic review" and protocols were used. The search from the above databases confirmed that there was no systematic review and /or protocol on the topic of interest.

| Serial number | Databases | Number of article found | Number of article included | Number of Excluded article | Reason for exclusion |
| --- | --- | --- | --- | --- | --- |
| 1 | PubMed | n=53 | n=17 | n=36 | duplicates |
| 2 | Google Scholar | n=60 | n=7 | n=53 | duplicates |
| 3 | Web of Science | n=27 | n=2 | n=25 | duplicates |
| 4 | Cochran Library | n=10 | n=0 | n=10 | Irrelevant papers |
| 5 | Africa Wide Knowledge | n=22 | n=0 | n=22 | duplicates |
| 6 | World Health Organization (WHO) afro library | n=29 | n=2 | n=27 | Irrelevant papers |
| 7 | Scopus | n=24 | n=4 | n=20 | Irrelevant papers |
| 8 | Africa Index Medicus | n=3 | n=1 | n=2 | Irrelevant papers |
| 9 | Microsoft Academic | n=7 | n=0 | n=7 | Irrelevant papers |
| 10 | Unpublished (pre-prent, manuscript, thesis and report from WHO) | n=10 | n=1 | n=9 | Irrelevant papers |

The included databases and number of included studies thereof were PubMed (53), Scopus (24), Google Scholar (60), and the World Health Organization (WHO) afro library (29). Of these studies, 101 duplicates were identified and removed. Subsequently, we screened 144 titles and abstracts and excluded 66 irrelevant papers. Then, based on the pre-defined criteria and quality assessment, 34 full-text articles with 13086 total diabetic patients were included in this systematic review and meta-analysis.
